# Method to evaluate the microbial degradation activity in silage, cow rumen with *in vitro* test, and in manure and slurry

**DOI:** 10.1016/j.mex.2024.102550

**Published:** 2024-01-05

**Authors:** Nebojša Nikolić, Selene Massaro, Franco Tagliapietra, Andrea Squartini, Stefano Schiavon, Roberta Masin

**Affiliations:** Department of Agronomy, Food, Natural Resources, Animals and Environment, University of Padova, 35020 Legnaro, PD, Italy

**Keywords:** Microbial activity, Proteolytic microorganism, Cellulolytic microorganism, Fertimetro

## Abstract

Microorganisms are present everywhere and can influence a variety of processes. In agriculture and husbandry, the level of microbial activity can be crucial information, yet the methods for determining microbial activity are usually very long, complex, and costly. In this work, a novel and easy-to-use method, already in use for determining soil microbial activity, named Fertimetro was tested as a fast and cheap solution for measuring microbial activity in silages, in vitro rumen fluids, and manure and slurry. The method was adjusted for the specific conditions of the new testing environments. The results indicate that this method is adequate for measuring cellulolytic microbial activity in vitro rumen fluids, with a coefficient of repeatability (RT%) 92.2 at 24 h and 87.5 at 48 h, and also for cellulolytic microbial activity measures in manure RT% 39.0. While, due to the specific conditions in silages and slurry, this method is less adequate for measuring cellulolytic microbial activity in these environments. This work demonstrates that Fertimetro method can be used in different environments as an easy and cheaper alternative for measuring microbial activity, especially if the interest is only in quantifying the microbial activity and not in knowing the microbial species.

1. Fertimetro is an easy-to-use and not costly method to evaluate microbial activity in different environments.
2. This method is very adequate for measuring cellulolytic microbial activity in vitro rumen fluids and manure.

## Graphical abstract

**Figure:**
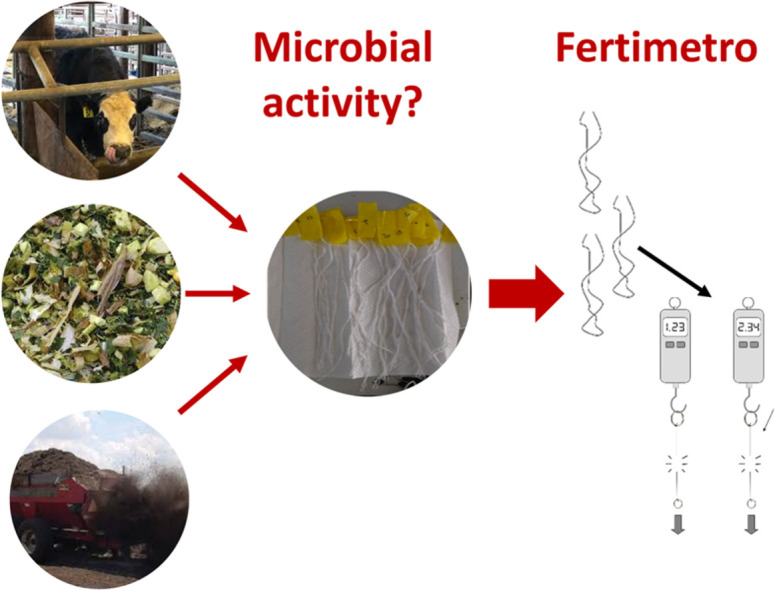

Specifications table

**Table:** 

Subject area:	Agricultural and Biological Sciences
More specific subject area:	Microbial degradation activity
Name of your method:	Fertimetro
Name and reference of original method:	Concheri, G., Tiozzo, S., Stevanato, P., Morari, F., Berti, A., Polese, R., et al. (2018a). Fertimetro, a Principle and Device to Measure Soil Nutrient Availability for Plants by Microbial Degradation Rates on Differently-Spiked Buried Threads. *Soil Syst.* 3, 3. doi: 10.3390/soilsystems3010003. Concheri, G., Tiozzo, S., Stevanato, P., and Squartini, A. (2018b). The nutrient-primed incremented substrate degradation principle . A novel method and an automated tool to assess and correct agricultural soil de fi ciencies to optimize its fertility and crop productivity. *Appl. Soil Ecol.* 123, 686–692. doi: 10.1016/j.apsoil.2017.07.001. (PCT/IB2012/001,157: Squartini, Concheri, Tiozzo, University of Padova
Resource availability:	https://patents.google.com/patent/WO2012140523A1/zh

## Method details

 

## Introduction

Microorganisms play a critical role in the degradation of organic and inorganic compounds, exerting their influence in diverse environments, such as soil, animal digestive systems, and waste management systems [1], [2], [3], [4], [5], [6], [7]. Understanding and quantifying microbial activity in these contexts is of utmost importance for assessing ecological processes, optimizing agricultural practices, and monitoring environmental health. However, existing methods for measuring microbial activity often present limitations, such as high costs, specialized training requirements, complex equipment, and the generation of excessive data or ambiguous interpretations. Additionally, accurately differentiating between microbial numbers and their actual activity poses a challenge [8], [9], [10], [11], [12].

To address these issues, the development of a simplified and cost-effective method called the Fertimetro [13], has gained attention. This innovative approach offers a practical solution for assessing cellulolytic and proteolytic microbial activity, which are key indicators of organic matter degradation. The Fertimetro method is based on the measurement of thread resistance against degradation by microorganisms, specifically utilizing treated cotton threads to assess cellulolytic activity and silk threads for proteolytic activity. By comparing the resistance of these treated threads with untreated ones, the method provides direct and easy-to-interpret results that reflect the biodegradative potential of microbial communities (PCT n. WO2012140523A1, [14]).

Initially designed for assessing microbial activity in soil ecosystems, the Fertimetro method's simplicity and potential broader applications have sparked interest in exploring its utility in other environments.

This study aims to investigate the feasibility of employing the Fertimetro method to measure cellulolytic and proteolytic activity in several distinct settings: silage, cow rumen (in vitro), manure, and slurry. These environments represent critical stages in the cycle of organic matter transformation, from plant material preservation through ensiling to livestock digestion and waste management [15], [16], [17], [18], [19], [20], [21], [22]. By focusing on these specific contexts, we aim to provide a comprehensive understanding of the method's applicability and reliability across various microbial activity assessment scenarios.

The outcomes of this study have significant implications for both scientific research and practical applications. If proven effective, the Fertimetro method could serve as a valuable tool for researchers, farmers, and environmental managers to evaluate microbial activity with ease and accuracy. Furthermore, its affordability and simplicity make it accessible to a wider range of users, including non-academic practitioners who require reliable microbial activity measurements without the need for extensive resources or specialized expertise. By emphasizing the development and application of the Fertimetro method, this research contributes to advancing the field of microbial ecology and offers practical solutions for enhancing sustainability and productivity in diverse ecosystems.

## Materials and methods

### Threads preparation and resistance measurement

Cotton and silk threads (10 cm long) were used in all the following trials. Both threads are commercially available and were purchased from Cucirini Tre Stelle s.r.l. (Caleppio di Settala, Milan, Italy). In order to replicate the original methods, the same type of threads was used as in Concheri et al. [13], that was n° 16 cotton thread and “Bozzolo Reale” (Royal Cocoon) n° 24 silk thread. As a necessary adjustment to the specific conditions in the environments tested, for all trials, the threads of both types were cut to 10 cm length and placed inside woven nylon bags with 50 ± 10 µm porosity by Ankom (ANKOM Technology^Ⓡ^, Macedon, NY, USA). These bags have already been used for different analyses. Their porosity allows the passage of all microorganisms (especially in the in vitro rumen) but maintains the material studied secured inside [23]. The threads were placed inside bags (5 cm x 5 cm size) and the top of each bag was sewn to keep the threads inside. The use of these bags allowed the optimal performance of the trials, without the risk of thread loss. Each bag contains six cotton or silk threads, for each trial, two bags per thread type were used, meaning that for each trial there were 12 measurements of cotton and 12 measurements of silk threads resistance. In order to achieve data robustness, each trial was repeated three times. After exposure to microbial activity in different trials, the bags with the threads were retrieved, opened, and the threads extracted and left to dry. Once the threads were dry, their resistance to breakage was measured using a digital dynamometer (IMADA ZP, ELIS Electronic Instruments and Systems, Rome, Italy). Each thread was attached to a keyring by pinching the end in and coiling the thread several times around the ring. Before measuring, the “peak” function mode of the dynamometer was selected, as it expresses the maximum load withstood by the thread before breaking. This peak force was then measured by pulling the ends of the tread until breaking them. Additionally, non-treated cotton and silk threads were measured as standards for determining the increase in treated threads' fragility. Data obtained from the measurements of the treated threads were compared to the average values of the respective standard.

### Silage trials

The ensiling process was conducted at the experimental farm "Lucio Toniolo" of the University of Padova, situated at Legnaro, Padova province. For the silage trials, three different types of silage were used: corn (*Zea mays*), sorghum (*Sorghum bicolor*) and a mix of grasses (50 % wheat (*Triticum aestivum*), 16 % triticale (*× Triticosecale*), 12 % Proteinic pea (*Pisum sativum*), 12 % Common vetch (*Vicia sativa*) and 10 % oats (*Avena sativa*)). The silage was made into round bales closed with nylon and stored. For this trial, two round bales of each type of silage were used, the trials took place several months after the ensiling process when all of the fermentation process was presumably stabilized. Following the experimental design, two bags with cotton and two bags with silk threads were prepared for each silage type. As two bales of each silage type were used, half of the bags prepared were placed in one bale and half in the other. The experimental design was: 3 silages x 2 bales x 6 cotton or silk threads x 3 repetitions, for a total of 108 measures for each kind of thread) The bags with threads were placed inside the bales by making a small incision which was immediately sealed after the insertion of the bags. The bags remained inside the silage bales for one week. After one week, the seals were broken and the bags retrieved, the threads were taken out and air-dried, and then their resistance to breakage was measured as described previously.

### In vitro rumen trials

Artificial rumen Daisy II (ANKOM Technology^Ⓡ^, Macedon, NY, USA) was used to test the method to determine the microbial activity inside the bovine rumen. This artificial rumen consists of a main box in which four bottles are placed, the bottles turn thanks to the small wheels installed on the bottles sloth and the temperature can be regulated, simulating the conditions inside the rumen. For this experiment, two different diets were tested, one for lactating cows and one for heifers, using two bottles for the first and two for the second diet. Microbial activity was monitored after 12, 24 and 48 h of thread incubation. As for the previous trial, following the experimental design, two bags with 6 cotton threads and two bags with 6 silk threads were prepared for each incubation time - diet combination. Considering that two bottles were used for each diet, half of the bags prepared for each incubation time - diet combination were placed in one bottle and half in the other. For each kind of thread, the experimental design was: 2 diets x 2 bottles x 6 threads x 3 times, for a total of 72 measures. The temperature of the instrument was set at 39 °C to heat the bottles and the diet before the insertion of the rumen fluid. The medium solution was prepared on the day of the trials, according to Menke and Steingass [24]. A total of 2-L rumen fluid was collected from two fasting Simmental lactating cows and two fasting Simmental heifers from the experimental farm “Lucio Toniolo” of the University of Padova (Legnaro, Italy) on the morning of the fermentation. The rumen fluid was collected from the animals and maintained in anaerobic conditions in a thermos to be transported to the laboratory. Then, the rumen fluid and medium solution (ratio 1:2) mixture was incubated in each bottle (800 mL of mixed rumen fluid + 1600 mL of medium) for 48 h. At the start of the experiment, in each bottle, of the specific diet, 12.5 gs of diet were weighed, both for the lactating cows and the heifers, to support the microbial fermentations and simulate the rumen conditions. Throughout the experiment, the 4 bottles were kept at 39 °C, avoiding opening the instrument door, and were rotated on themselves to simulate ruminal movements in the animal. At 12, 24, and 48 h, each bottle was opened and the bags containing the threads were removed. Then, threads were taken out and passed under the jet of warm water to block the microbial activity, then air dried before measuring the thread resistance as described above.

### Manure and slurry trials

To test the applicability of this method to measure the cellulolytic and proteolytic microbial activity in manure and slurry, a trial site was set up at the experimental farm of the University of Padova. A part of the manure produced by the cows was retrieved and placed separately, the slurry was collected and placed in a container 1 m^3^ in volume to be easily accessible. Two bags with cotton and two bags with silk threads were prepared for each measurement in both manure and slurry, in order to retrieve the bags more easily, containers were made from plastic nets with 1 mm porosity and the bags were placed inside. The containers were afterward anchored with strings to help the retrieval. A weight was also placed inside the containers for the slurry trial to keep the threads submerged. The bags were placed inside manure and slurry at the same time and were left there for a week, after which they were retrieved, washed under warm water, air-dried and subsequently measured as explained previously. As all other trials, this experiment was repeated three times, but in different seasons mid-July 2022, at the start of September 2022 and mid-October 2022. In both slurry and manure, the temperature was measured using LPWAN miniUNI PT100 probes (Solidus TECH s.r.o., Frýdek-Místek, Czech Republic). For each kind of thread, the experimental design was: 2 mediums x 12 threads x 3 seasons, for a total of 72 measures.

### Statistical analysis

The value of the resistance to the breaking of the threads was calculated with the following formula:

(1) R=(Ri/Rni)×100

where R is the resistance percentage; Ri is the rupture value of the threads exposed to microbial activity, and Rni is the rupture value of the new (untreated) filament.

(2) D=100−R

In order to express the value as biological activity, the resistance percentage was converted into a degradation percentage (D) by subtracting resistance percentage values from 100 [13].

Data obtained from the threads measurements were statistically analysed using factorial ANOVA, after performing the Leven test, followed by Tukey post-hoc test. All statistical analysis were performed inside the R environment [25].

To estimate the repeatability of the measures, eestimation of variance components was accomplished separately for estimates of cotton threads degradation (D,%) in silages, in rumen fluid for 12, 24 and 48 h, and in manure or slurry using the PROC MIXED of SAS (2005) with the following mixed linear models:

- for cotton threads degradability in silages was considered as random effects the silage type (S), the repetition (R1), the interaction S x R1 and the error term (e1).
- for cotton threads degradability in rumen fluid (at 12, 24 and 48 h) was considered as random effects the rumen fluid (RF), the repetition (R2), the interaction RF x R2 and the error term (e2).
- for cotton threads degradability in waste (manure or slurry) was considered as random effects only the repetition (R3) and the error term (e3).

The restricted maximum likelihood method (REML) was used as the method of estimation of variance components. For each model, the components of variance of each factor were used to compute the repeatability (RT), defined as the value below which the absolute difference between two single measures obtained with the same method and under the same conditions (i.e., same silage, same diet) is expected with a 95 % probability, and the coefficient of repeatability (RT%) and coefficient of variation (CV) [26,27], as:

$$\text{Silage}\;\text{trial}:RT_{\text{silage}}=2\surd 2\sigma_{e1}^{2}$$

$$\;\;\;CV_{\text{silage}}=\frac{RT_{\text{silage}}}{\text{Mean}}\times \;100$$

$$\;\;\;\text{RT}{\%}_{\text{silage}}=\frac{\sigma_{S}^{2}+{\;\sigma }_{R1}^{2}+\sigma_{\text{SxR}1}^{2}}{\sigma_{S}^{2}+{\;\sigma }_{R1}^{2}+\sigma_{\text{SxR}1}^{2}+\sigma_{e1}^{2}}\times \;100$$

$$\text{Rumen}\;\text{fluid}\;\text{trial}:\;RT_{\text{at}\;12,\;24\;\text{or}\;48h}=2\surd 2\sigma_{e2}^{2}$$

$$\;\;\;CV_{\text{at}\;12,24\;\text{or}\;48\;h}=\frac{RT_{\text{at}\;12,24\;\text{or}\;48h}}{\text{Mean}}\times \;100$$

$$\;\;\;\text{RT}{\%}_{\text{at}\;12,\;24\;\text{or}\;48h}=\frac{\sigma_{\text{RF}}^{2}+{\;\sigma }_{R2}^{2}+\sigma_{\text{RFxR}2}^{2}}{\sigma_{\text{RF}}^{2}+{\;\sigma }_{R2}^{2}+\sigma_{\text{RFxR}2}^{2}+{\;\sigma }_{e2}^{2}}\times \;100$$

$$\text{Waste}\;\text{trial}:\;RT_{\text{manure}\;\text{or}\;\text{slurry}}=2\surd 2\sigma_{e3}^{2}$$

$$\;\;\;CV_{\text{manure}\;\text{or}\;\text{slurry}}=\frac{\text{manure}\;\text{or}\;\text{slurry}}{\text{Mean}}\times \;100$$

$$\;\;\;\text{RT}{\%}_{\text{manure}\;\text{or}\;\text{slurry}}=\frac{\sigma_{R3}^{2}}{\sigma_{R3}^{2}+{\;\sigma }_{e3}^{2}}\times \;100$$

## Method validation

### Silage microbial activity

For both silk and cotton threads, there were no significant differences in degradation, both for interactions and for single factors (Tables 3 and 4).

There was little to no degradation by both types of microorganisms, ranging from 5.04 ± 1.82% to 11.93 ± 2.09% degradation for cotton threads and from 0 to 5.58 ± 2.38% for silk threads. However, this result is not surprising considering the conditions inside the silage, already mentioned before [17,28]. The small levels of degradation could be attributed to the acidic condition of the silage (pH 4,1 °C), to the prevailing lack of oxygen to support an efficient heterotrophic metabolism given the brief exposure of silage to aerobic conditions limited to the stage of placing the threads inside. In fact, as the silage bales were immediately sealed, the microbial activity can be considered to have stopped after all the air was used [17,18,[28], [29], [30]]. The results obtained from this trial are in fact in line with their expected outcome. That is, low to no cellulolytic and proteolytic microorganism activity due to specific conditions in the silage. As previously said, silage is made for prolonged conservation, therefore a minimal microbial activity is observed when the ensiling process is conducted properly.

### In vitro rumen microbial activity

As for the other trials, results from silk threads measurement showed no statistically significant differences and the degradation percentage remained very low (Table 5).

Results obtained from the measurement of the silk threads indicate that these threads are not an adequate solution for measuring the proteolytic activity inside the rumen.

Considering that silk fibers have a compact, regular and hydrophobic structure, the short residence times of the fibers in the rumen and the fermentative conditions do not allow the degradation of this type of protein.

On the other hand, the results from the cotton threads measurement have shown very interesting and significant results (Table 6).

Statistical analysis indicates that the only significant interaction is between the incubation time (12, 24 and 48 h) and the rumen fluid donor (lactating cows and heifers).

**Fig. 1:**
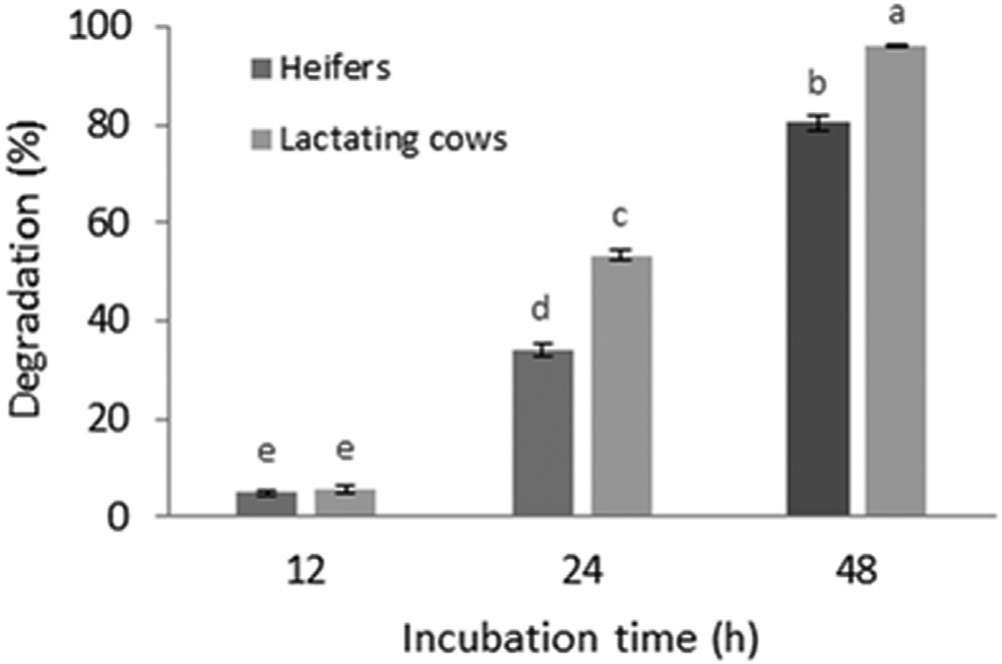
Degradation percentage of cotton threads exposed to different rumen fluids and to different incubation times. Different letters indicate a significant difference (*p* < 0.05).

The microbial activity increased with time, showing almost complete degradation after 48 h of exposure, which is in agreement with what was found by Wang and Duan [31] (Fig. 1). As different authors indicate, cellulolytic microorganisms' activity also depends on the feed ingested [32], [33], [34], which might explain the results obtained in this trial where the animal received different diets (Table 1), with different proportions of cellulose, hemicellulose and lignin (Table 2). It is possible to observe that, after 12 h of incubation, there were statistically significant differences in threads degradation between the two rumen fluids, as the fluid collected from lactating cows showed higher microbial activity compared to that of heifers. Although this may seem odd, it is in agreement with what was discovered by Nikolić et al. [35], in the experiments with soil, where cotton threads were degraded more in soils with less cellulose content compared to the ones with higher cellulose content. The authors attributed this effect to the fact that the microorganisms are less prone to attack the additional cellulose in a highly saturated environment, continuing to biodegrade the one already present.

**Table 1:** Composition of the two diets used in the experiment, the values are expressed as% of DM.

Material (% DM)	Lactating cows	Heifers
Corn silage	20.1	–
Grass silage	3.8	15.5
Sorghum silage	31.5	6.2
Alfalfa hay	6.0	–
Ryegrass hay	–	47.0
Corn Gluten feed	6.1	16.0
Energy mixture (corn-barley)	11.7	–
Protein mixture (soy, sunflower)	16.1	–
Soybean meal	–	13.5
Vit-Mineral mix and wheat/flax germ	1.3	–
Vitamin supplement	**–**	1.8
Molasses	3.4	–
Total	100	100

**Table 2:** Nutritional composition of the rations used for the tests (% DM).

Content	Lactating cows	Heifers
Dry Matter (%)	51.2	74.9
Hemicellulose	16.0	19.6
Cellulose	22.9	24.1
Lignin	3.3	3.2
Protein	15.7	12.5
Fats	3.5	1.5
Starch	24.2	4.4
Ashes	5.52	8.03

**Table 3:** Full factorial ANOVA on the degradation of cotton threads in silage.

Factors	Df	F-value	p-value
Repetition	2	2.464	0.090
Silage type	2	1.230	0.297
Repetition x Silage	4	0.398	0.809

ns- Not significant; Df- Degrees of freedom.

**Table 4:** Full factorial ANOVA on the degradation of silk threads in silage.

Factors	Df	F-value	*p*-value
Repetition	2	2.735	0.070
Silage type	2	1.904	0.154
Repetition x Silage	4	2.000	0.100

ns- Not significant; Df- Degrees of freedom.

**Table 5:** Full factorial ANOVA on the degradation of silk threads in rumen.

Factors	Df	F-value	*p*-value
Repetition	2	0.924	0.401
Incubation time	2	1.039	0.358
Diet	1	0.324	0.571
Repetition x Incubation time	4	1.040	0.391
Repetition x Diet	2	2.151	0.122
Incubation time x Diet	2	0.213	0.809
Repetition x Incubation time x Diet	4	0.858	0.492

ns- Not significant; Df- Degrees of freedom.

**Table 6:** Full factorial ANOVA on the degradation of cotton threads in rumen.

Factors	Df	F-value	*p*-value
Repetition	2	0.4321	0.651
Incubation time	2	4382.7334	< 2 ^−^ ^16^
Rumen fluid	1	268.8543	< 2 ^−^ ^16^
Repetition x Incubation time	4	1.8398	0.128
Repetition x Rumen fluid	2	3.0433	0.053
Incubation time x Rumen fluid	2	62.9541	< 2 ^−^ ^16^
Repetition x Incubation time x Rumen fluid	4	1.5288	0.201

ns- Not significant; Df- Degrees of freedom.

Differently from the proteolytic microorganisms, it was possible to observe the activity of cellulolytic microorganisms. This is not surprising, considering that the rumen, in spite of its anoxic condition, is recognized as a natural bioreactor for cellulose, hemicellulose and lignin degradation through efficient fermentative co-metabolism [36]. Rumen includes many cellulose-degrading microorganisms, primarily bacteria, fungi, and protozoa [37], [38], [39], [40], [41], [42]. These microorganisms can degrade cotton fibers which are made of pure cellulose, and indeed attempts to measure the cellulolytic microbial activity using these threads were already performed in the past [33]. Stewart [33], used the sheep ruminal liquid and exposed the threads to microbial activity for 24 h. He concluded that this method is more efficient and reliable than other methods, such as the test with filter paper strips. Differently from Stewart [33], in our work, the ruminal liquids were incubated for three different times, assessing the possibilities that the ingested material can remain in the rumen for different times but that it is mainly degraded after 48 h [31]. However, our findings concur with the conclusions of Stewart [33], about the suitability of this method for determining cellulolytic microbial activity.

### Microbial activity in manure and slurry

Microbial activity was very different between manure and slurry, especially the cellulolytic microbial activity (Table 7).

**Table 7:** Full factorial ANOVA on the degradation of cotton threads in manure and slurry.

Factors	Df	F-value	*p*-value
Season	2	3.0557	0.054
Medium	1	254.677	< 2.2 ^−^ ^16^
Season x Medium	2	11.9603	3.7 ^−^ ^05^

ns- Not significant; Df- Degrees of freedom.

**Fig. 2:**
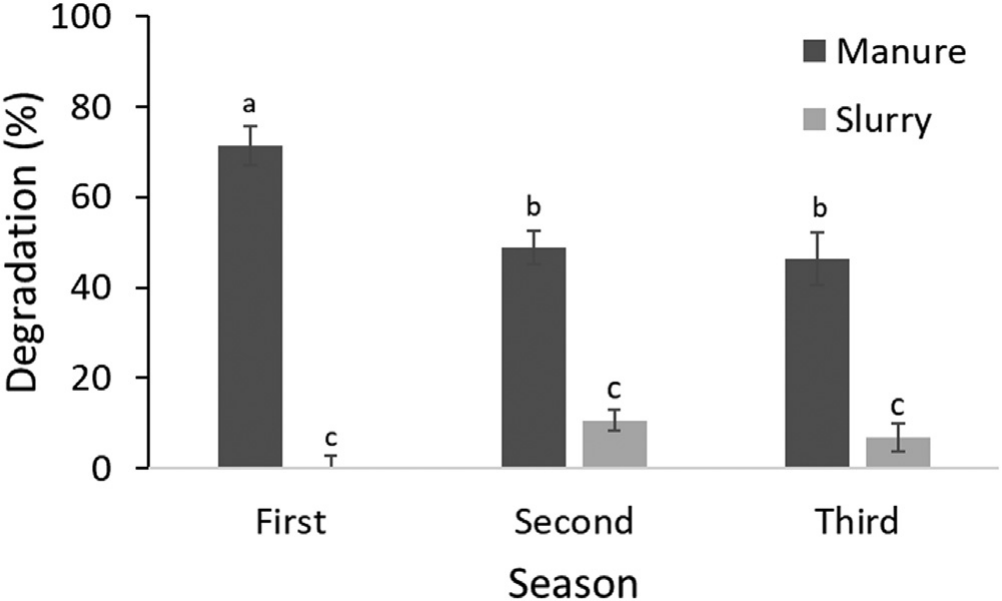
Degradation percentage of cotton threads exposed to microbial activity in manure and slurry during different seasons. Different letters indicate a significant difference (*p* < 0.05).

Statistical analysis indicates a significant difference between the two mediums tested (manure and slurry) and the interaction between the mediums and different repetitions (Fig. 2).

**Fig. 3:**
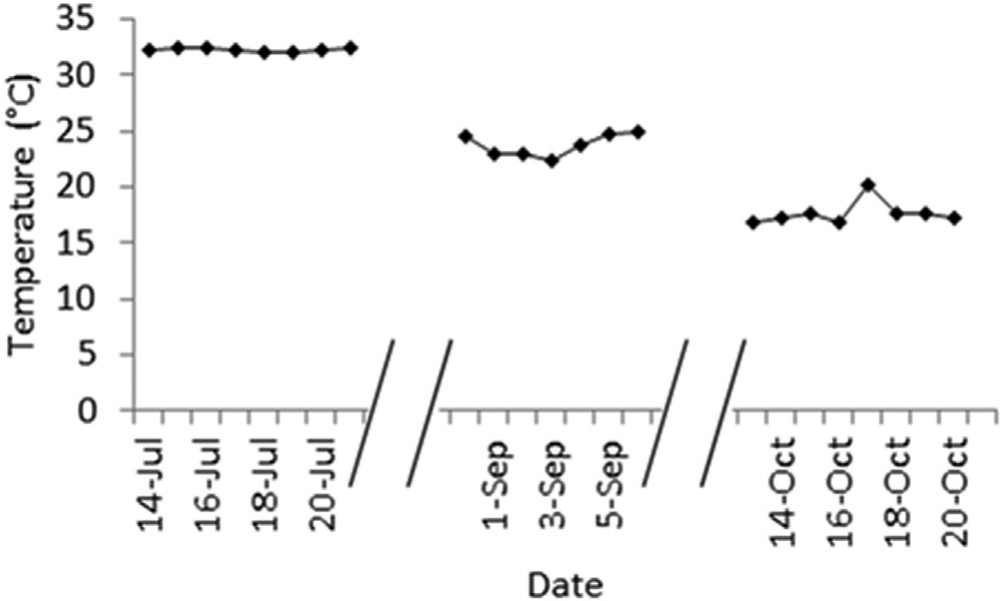
Mean daily temperatures recorded in manure during the three experiment periods.

There were no differences in threads degradation in slurry, and the degradation levels remained very low, indicating a low cellulolytic microbial activity (Fig. 2). This result is probably due to the slurry anaerobic conditions that have prevented cellulolytic microorganisms' activity [43]. Differently from the slurry, high microbial activity was observed in manure, and the first repetition differed from the others. These findings can be explained by the higher temperature recorded in manure during the first trial compared to the last two (Fig. 3). In fact, it is known that temperature plays an important role in microbial activity [44,45].

The slurry substrate is expectedly also more limiting degradation activities due to its liquid composition which is the strongest barrier to oxygen diffusion. Furthermore, microbial activity is usually very high in cattle manure, comprising several aerobic microorganisms, such as fungi and bacteria [46,47]. Structural changes can also be observed during the composting of the manure, which allow major air penetration and the consequent change in microorganisms' activity [48]. Our findings confirm that the Fertimetro method is adequate for measuring manure's cellulolytic microbial activity.

As for the activity of proteolytic microorganisms, the degradation of silk threads remained very low, with the only significant factor being the repetition of the trials (Table 8).

**Table 8:** Full factorial ANOVA on the degradation of silk threads in manure and slurry.

Factors	Df	F-value	*p*-Value
Season	2	20.3782	1.28^−07^
Medium	1	0.0877	0.768
Season x Medium	2	1.1832	0.313

ns- Not significant; Df- Degrees of freedom.

**Fig. 4:**
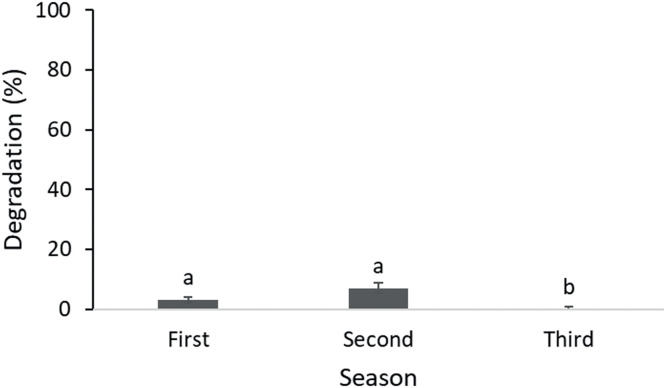
Degradation percentage of silk threads exposed to microbial activity in manure and slurry during different seasons. Different letters indicate a significant difference (*p* < 0.05).

Although it shows a significant difference, the degradation percentages were extremely low (Fig. 4), raising concerns as to whether the degradation found might be more a result of physical and/or chemical degradation rather than microbial. Results about the silk threads degradation from the experiment with manure and slurry add to the idea that silk threads are not an adequate material for measuring proteolytic microbial activity in these environments.

### Method repeatability

Considering the low level of silk threads degradation, repeatability examinations of the method presented focused only on the use of cotton threads. Results obtained for all the mediums tested in this work can be seen in Table 9, Table 10, Table 11.

**Table 9:** Mean cotton threads degradability (D%) and coefficients of repeatability measured in silages.

	Degradability
Mean	7.5
RT	19.7
CV	262.4
RT%	4.7

**Table 10:** Mean cotton threads degradability (D%) and coefficients of repeatability measured after 12, 24 and 48 h of fermentation in rumen fluid.

	Degradability		
	12 h	24 h	48 h
Mean	5.2	43.6	88.3
RT	6.9	12.8	12.0
CV	134.0	26.0	13.5
RT%	1.6	92.2	87.5

**Table 11:** Mean cotton threads degradability (D%) and coefficients of repeatability measured in manure and slurry.

	Manure	Slurry
Mean	55.5	5.9
RT	45.9	19.7
CV	82.8	335.8
RT%	39.0	0.7

As evidenced by the results, the method for detecting the degradation of cotton threads using various media demonstrates a wide range of values for both repeatability (RT) and the coefficient of repeatability (RT%). Specifically, both RT and RT% are lower for silages and slurry, where low microbial activity was measured. On the other hand, the values for manure are much higher, suggesting that this method can be used for fast and undemanding measurement of microbial activity. Similar trend is noticeable in the measurements of rumen microbial activity. Both RT and RT% are very low for the 12 h test, whereas they are considerably higher for the 24 h and 48 h tests. This suggests that the microbial activity with the Fertimetro method should preferably be tested at 24 h or 48 h. Interestingly, both RT and RT% are higher at 24 h compared to 48 h, which supports the idea presented by Tagliapietra et al., [49], that the testing duration for in vitro rumens studies can be reduced from 48 h to 24 h. Considering this, it can be asserted that an additional advantage of Fertimetro method could also be the reduced time needed for measuring the microbial activity.

## Conclusions

Considering the results obtained, it is possible to state that the technology of the Fertimetro method can be transferred from soil to other environments/mediums. The Fertimetro method offers an easier and cheaper alternative for measuring microbial activity compared to other available methods, especially considering that the investment mostly includes cotton and silk threads. This method is especially suitable if the interest is only in quantifying the microbial activity and the biodegradation caused by microorganisms and not in knowing the microbial species. The authors’ opinion is that this method could find its application in different scientific and industrial areas as a cheap explorative method of microbial activity. However, based on the results, it is possible to conclude that this method is reliable for measuring only the cellulolytic microbial activity, considering that little to no degradation was found for silk threads. Indeed, none of the silk threads trials showed any statistical difference, and the general degradation of silk threads was extremely low. These findings would indicate that unlike the soil [14,13], silk threads, featuring proteins as sericin and fibroin, are not an adequate material for measuring proteolytic microbial activity inside the specific environments hereby tested in which the corresponding enzymatic capabilities might not be sufficiently represented as in other more open environments. Further trials are necessary to determine the validity of this method for measuring microbial activity in different or similar environments, such as, for example, different types of manure and different ruminal liquids. Moreover, it is important to find a substitution for silk threads to have a reliable option for measuring the proteolytic microbial activity.

## CRediT authorship contribution statement

**Nebojša Nikolić:** Conceptualization, Data curation, Formal analysis, Investigation, Methodology, Resources, Software, Writing – original draft. **Selene Massaro:** Data curation, Investigation, Methodology, Resources, Writing – review & editing. **Franco Tagliapietra:** Conceptualization, Data curation, Formal analysis, Resources, Software, Writing – review & editing. **Andrea Squartini:** Conceptualization, Data curation, Formal analysis, Resources, Software, Writing – review & editing. **Stefano Schiavon:** Conceptualization, Supervision, Visualization, Writing – review & editing. **Roberta Masin:** Conceptualization, Data curation, Formal analysis, Methodology, Supervision, Visualization, Writing – review & editing.

## Declaration of Competing Interest

The authors declare that they have no known competing financial interests or personal relationships that could have appeared to influence the work reported in this paper.

## Data Availability

Data will be made available on request.
